# Disease identification based on ambulatory drugs dispensation and in-hospital ICD-10 diagnoses: a comparison

**DOI:** 10.1186/1472-6963-13-453

**Published:** 2013-10-31

**Authors:** Patricia Halfon, Yves Eggli, Anne Decollogny, Erol Seker

**Affiliations:** 1 Institute of Social and Preventive Medicine (IUMSP), University Hospital Center and Faculty of Biology and Medicine, Biopole 2, Route de la Corniche 10, 1010, Lausanne, Switzerland; 2 Institute of Health Economics and Management, University Hospital Center and University of Lausanne, Route de Chavannes 31, 1015, Lausanne, Switzerland

**Keywords:** Case mix, Pharmacy data, Ambulatory care, Drug utilization, Kappa coefficients

## Abstract

**Background:**

Pharmacy-based case mix measures are an alternative source of information to the relatively scarce outpatient diagnoses data. But most published tools use national drug nomenclatures and offer no head-to-head comparisons between drugs-related and diagnoses-based categories. The objective of the study was to test the accuracy of drugs-based morbidity groups derived from the World Health Organization Anatomical Therapeutic Chemical Classification of drugs by checking them against diagnoses-based groups.

**Methods:**

We compared drugs-based categories with their diagnoses-based analogues using anonymous data on 108,915 individuals insured with one of four companies. They were followed throughout 2005 and 2006 and hospitalized at least once during this period. The agreement between the two approaches was measured by weighted kappa coefficients. The reproducibility of the drugs-based morbidity measure over the 2 years was assessed for all enrollees.

**Results:**

Eighty percent used a drug associated with at least one of the 60 morbidity categories derived from drugs dispensation. After accounting for inpatient under-coding, fifteen conditions agreed sufficiently with their diagnoses-based counterparts to be considered alternative strategies to diagnoses. In addition, they exhibited good reproducibility and allowed prevalence estimates in accordance with national estimates. For 22 conditions, drugs-based information identified accurately a subset of the population defined by diagnoses.

**Conclusions:**

Most categories provide insurers with health status information that could be exploited for healthcare expenditure prediction or ambulatory cost control, especially when ambulatory diagnoses are not available. However, due to insufficient concordance with their diagnoses-based analogues, their use for morbidity indicators is limited.

## Background

Building health indicators, managing health care and prevention, and adjusting for insurers’ risks require the assessment of morbidity burdens [1]. Demographic variables do not account sufficiently for the discrepancy in health service use and costs, overestimating cost variations between care providers and misidentifying outliers [2,3].

Most developed countries have minimal data sets on inpatient morbidity and causes of death. Outpatient morbidity information is scarcer except for cancer registers and contagious infections, which are subject to mandatory declaration. National health surveys have been conducted to estimate the prevalence of chronic illnesses but such expensive and time-consuming studies are generally not feasible on an ongoing basis [4,5]. Although the increased use of electronic medical records (EMR) by primary physicians has the potential to collect clinical information in large populations, the identification of a particular disease within an EMR often remains far from straightforward [6,7].

Current patient classification systems are mainly based on diagnoses information. In the USA, Medicare and Medicaid databases and some private health insurance or maintenance organizations routinely record ambulatory diagnoses. In Switzerland, as in many other countries, such records are missing mainly because data collection is time-consuming, costly and not always reliable [8,9].

Thence the growing interest in measures based on drug prescription data, often routinely collected by insurers; they may also provide information on well-controlled diseases, which are frequently under-declared by physicians [10,11].

Most medication-based classification systems are derived from the chronic disease score (CDS) developed by Von Korff et al., with a fair prediction of hospitalization, mortality, the number of ambulatory visits and costs [12-14]. Improvements now include a wider range of drugs, new scores, and extended application to various populations (pediatric, Medicare and Medicaid, veterans, European countries) [15-18]. For example the “Rxrisk” model developed by Fishmann included 55 therapeutic categories. It was designed to predict future health costs and thus restricted to chronic diseases [19].

Only a few studies on selected populations have checked criterion validity by comparing drugs categories head-to-head with their diagnoses-based analogues [18]. As measured by the Kappa coefficient (< 0.4), 40% of the “Rxrisk” categories seldom matched with their ICD-9-CM based counterparts. Drug rates provided a valid estimation of diagnosed and treated prevalence for only few medical conditions [20,21].

Many drugs- related classification systems were built on national drug nomenclatures [14,17]. However, since indications for certain agents differ depending on how they are administered, names alone do not adequately express a condition. Pharmacy-based models should be regularly updated and validated to verify that they are not sensitive to practice variations.

The overall aim of our work was to develop a clinically relevant drugs-based case mix measure, derived from the WHO Anatomical Therapeutic Chemical (ATC) classification of drugs [22]. Diagnoses information not being available for ambulatory care, we limited the accuracy assessment of disease detection to the hospitalized population. Testing the performance of drugs-based patient classification systems to predict ambulatory resources or health outcomes was beyond the scope of our work.

## Methods

### Setting

Our study is an observational study based on routine data from four Swiss health insurers on approximately 2.0 million insured enrollees of whom 1.7 million were followed in 2005 and 2006. Among the latter, all insured hospitalized at least once in a Swiss hospital were retained. Data were collected with the support of the Swiss Federal Office of Public Health [23]. Dispensed medication was identified by a national product code (pharmacode). As in Switzerland pharmacists systematically send drugs codes and dispensation dates to insurers for billing purposes, we expect only minimal inaccuracies in those data. All Swiss citizens are covered by compulsory insurance and thus have unrestricted access to drugs. Few drugs dispensed in outpatient hospital settings (e.g. anti-neoplastic) were not in the database. Pharmacy data were linked to corresponding hospital diagnoses codes (ICD-10) [24] via the anonymous linkage code procedure by the Swiss Federal Statistical Office (SFSO); only a sequential number was delivered [25]. Hospital data supplied by the Federal Statistical Office (inpatient diagnoses) are publicly available. Insurers’ data (dispensed drugs) are not publicly available and were supplied only for the research project supported by the Federal Public Health Office, with the prerequisite of using the anonymous linkage code procedure of the Federal Statistical Office. All data were anonymous and did not include any element, which might allow identifying a single person (no birth date or ZIP codes for instance) [26]**.** More than 99% of the insured had a corresponding anonymous linkage code in the hospital SFSO data base. As in most other developed countries, only diagnoses which had an impact on the treatment of the patient were collected [27]. The space for recording diagnoses was limited to ten codes. These medical records were mainly used for epidemiological studies and hospital resources allocation (Diagnosis Related Groups). Several cantons allow physicians to dispense drugs to patients directly (self-dispensation). In such cases, information on dispensed drugs was limited to their costs. To avoid information bias, patients for whom self-dispensation represented more than 5% of drugs costs were excluded. For chronic diseases, all dispensed drugs were considered regardless of the dispensing and hospitalization dates, while for acute diseases only drugs dispensed two months before a hospital admission or after discharge were kept. In the event of several hospital stays of a same patient, all diagnoses and drugs related to each hospitalization were kept but the patient was considered as only one observation.

### Assigning diagnoses and drugs to morbidity groups

Diagnoses consisted of over 16,000 ICD10 codes, too many to be manageable. Therefore, in a first step, they were grouped under the 130 categories of the International Shortlist for Hospital Morbidity Tabulation (ISHMT) recommended by the World Health Organization [24]. Diagnoses groups which usually do not require a specific drug treatment (trauma, surgical or obstetrical conditions, congenital malformations, and unspecified morbidities or symptoms) were excluded (Additional file 1). Morbidity groups were deduced from main and secondary diagnoses coded in hospital medical records.

All dispensed drugs were attributed to the ATC classification system, after the exclusion of other pharmaceutical products (dressings, homeopathy, herbal medicine, etc.). We mainly used the therapeutic subgroups (2^nd^ level of the classification, e.g. A10 = anti-diabetic drugs), but a higher level of classification was sometimes required to identify a specific disease (e.g. therapy against HIV disease). Subgroups that could not be matched with any morbidity categories (blood products, anesthesia, etc.) were not taken into account (Additional file 1).

We identified all diagnostic categories assignable to one therapeutic subgroup. Most other diagnostic categories were subdivided to match therapeutic subgroups. In other cases, we grouped diagnostic categories requiring similar treatment. For certain conditions, we combined scattered ICD-10 codes corresponding to similar treatments (e.g. bacterial infection or thrombo-embolic diseases). Face validity of each morbidity category inferred from drug information was ensured by thorough reviews conducted by a skilled physician (PH) and a skilled pharmacist (AD) as regards the clinical homogeneity of the condition and the labeled use of the drug.

### Accuracy of morbidity groups identification

An algorithm identifying subjects with different conditions or diseases may be seen as a diagnostic test, and is generally assessed by the four estimates of diagnostic accuracy: sensitivity, specificity, positive and negative predictive values. These measures establish one of the classification procedures (here: morbidity categories based on inpatient ICD-10 codes) as a true gold standard. Yet we know that co-morbidities are often not recorded in hospital minimal data sets, especially if their impact on resource use is weak, or for patients with a more serious illness, where the severity of the condition and complications take precedence over chronic conditions in coding [28,29]. We thus focused mainly on the degree of agreement between the two morbidity classifications.

To ensure the identification of chronic diseases over both 2005 and 2006, a test-retest procedure was applied to all subjects classified in at least one drug-based category in 2005.

We computed the prevalence in 2005 of morbidities estimated from ambulatory drugs dispensation for all insured. Underestimation of cases due to the removal of subjects receiving medication directly from their physician was corrected via the assumption that morbidity distribution in this population was similar to the rest.

For comparisons against published estimates (external data), crude rates were standardized using gender and a distribution by five-year age categories of the Swiss population in 2005. When reference rates were available only for a specific population restricted to categories defined by age or sex, direct standardization of rates used only groups of this particular population.

### Statistical analysis

The results according to drug and diagnoses based information may be arranged in a four cells table with proportions as shown in Table 1.

**Table 1:** Agreement between diseases screened by drugs and hospital coded diagnoses

		**Hospital diagnoses**		
		**Condition present**	**Condition absent**	
Drug data	Condition present	a	b	Q
	Condition absent	c	d	Q´
		P	P´	1.0

The Kappa coefficient (K_c_), described by Cohen, is commonly used to assess agreement between two ratings [30]. It is obtained from the proportion of observed agreement, o = (a + d) and the expected agreement, e = (a + c) (a + b) + (c + d) (b + d), as follows.

$$K_{c}=\left(o-e\right)/\left(1-e\right)$$

Zero indicates only chance agreement, 1 indicates complete agreement beyond chance.

One limitation of K_c_ is that the measure ignores the relative utility of false positives (b) versus false negatives (c). To deal with this problem we proposed the weighted Kappa coefficient K_w,_ where the weight indicates the relative importance of false negatives versus false positives [31,32]. K_0_ (weight = 0) is recommended if expected false negatives (PQ’) have zero utility; K_1_ (weight = 1) if expected false positives (P’Q) have zero utility (see Additional file 1 for details).

Use of drug-based morbidities for measuring health care indicators was grounded on the values of the three Kappas, Κ_c_, Κ_0_, Κ_1_. Agreement between illnesses screened by delivered drugs and inpatient-coded diagnoses must be high to compare similar clinical situations. As suggested by Landis and Koch, we considered that a K_c_ over 0.4 describes a minimal level of agreement [33]. We relaxed this criterion if one of the weighted Kappas (K_0_ or K_1_) was respectively greater than 0.4 in the two following situations: existing alternatives to drug treatment explained a substantial proportion of false negatives (K_0_ >0.4 is required), or the high risk of under-reporting a non-severe morbidity explained a substantial proportion of false positives (K_1_ > 0.4 is required). We analyzed false positives and negatives in order to locate the potential errors in the two screening methods and - if possible – to improve the screening algorithm based on drug information.

In order to facilitate interpretation of the results, we chose to present the findings according to three potential fields of application of a heath status routine measure: morbidity indicators, ambulatory care cost control, and health insurers’ risk adjustment (Table 2, col. use). Morbidity indicators i.e., incidence or prevalence measures, (M in Table 2), require accurate disease detection, with K_C_ greater than 0.4, or K_1_ > 0.4, if false positives are explained by under-coded diagnoses. However, criteria can be relaxed to K_0_ or K_1_ > 0.2 for ambulatory cost control or risk adjustment (C and R in Table 2), since it is better to detect some morbidity rather than none. Note that only chronic diseases are relevant to insurers’ risk adjustment, given that the aim of that procedure is to forecast costs. Finally, despite satisfactory concordance, caution should be exercised when considering conditions for which the indication of drugs might be uncertain or is prone to practice variations (Table 2, footnote c).

**Table 2:** Accuracy of disease detection through drugs prescription among hospitalized patients (N = 108,915)

**Morbidity group**	**Diagnosed condition**	**Screened by drug**	**Sensitivity**	**Specificity**	**K**_**c**_^**a**^	**K**_**0**_^**a**^	**K**_**1**_^**a**^	**Use**^**b**^
**With K**_**C**_ **> 0.4**								
Transplant	467	601	0.82	1.00	0.71	(0.63)	(0.82)	M, C, R
Diabetes mellitus	8,171	8,114	0.71	0.98	0.70	(0.70)	(0.69)	M, C, R
HIV disease	413	372	0.60	1.00	0.63	(0.67)	(0.60)	M, C, R
Hypertensive disease	22,307	34,680	0.77	0.80	0.49	(0.37)	(0.67)	
*Alternative refinement:*								
Hypertension and heart failure	23,886	35,954	0.78	0.80	0.51	(0.39)	(0.68)	M, C, R
Thyroid disorders	3,179	2,993	0.40	0.98	0.40	(0.41)	(0.38)	M, C, R
**With K**_**0**_**>0.4 and alternatives to pharmacological treatment**								
Multiple sclerosis	444	115	0.20	1.00	(0.32)	0.78	(0.20)	C, R
Abuse of alcohol	4,584	619	0.09	1.00	(0.16)	0.68	(0.09)	C, R
Ischemic heart disease	10,691	4,537	0.26	0.98	(0.33)	0.56	(0.23)	C, R
Conduction disorders and cardiac arrhythmias	8,297	2,512	0.18	0.99	(0.26)	0.56	(0.16)	C, R
Malignant neoplasms	7,528	3,757	0.29	0.98	(0.36)	0.54	(0.26)	C, R
Abuse of opioid	1,131	45	0.02	1.00	(0.04)	0.51	(0.02)	C, R
Hepatitis B or C	885	90	0.05	1.00	(0.09)	0.50	(0.05)	C, R
Non-nutritional anaemia	6,114	642	0.05	1.00	(0.08)	0.41	(0.04)	
*Including those of end stage renal disease and due to chemotherapy*	8,868	642	0.05	1.00	(0.08)	0.63	(0.04)	C, R
**With K**_**1**_ **> 0.4**								
*Obvious under-coded diagnoses*								
Glaucoma	431	4167	0.70	0.96	(0.13)	(0.07)	0.69	M, C, R
Hyperlipidemia	8,052	18,050	0.71	0.88	(0.38)	(0.26)	0.65	M, C, R
Functional digestive disorders	1,275	27,846	0.63	0.75	(0.04)	(0.02)	0.50	^c^
Hyperplasia of prostate	2,793	7,816	0.52	0.94	(0.26)	(0.17)	0.49	M, C, R
Osteoporosis	2,443	4,765	0.49	0.97	(0.31)	(0.23)	0.46	M, C, R
*Signs of under-coded diagnoses*								
Mood disorders	6,649	19,624	0.68	0.85	(0.29)	(0.18)	0.61	M, C, R
Reactive airway disease	6,045	11,960	0.58	0.92	(0.36)	(0.25)	0.53	M, C, R
Alzheimer’s disease	844	1,307	0.48	0.99	(0.37)	(0.31)	0.48	M, C, R
Crohn’s disease and ulcerative colitis	433	824	0.48	0.99	(0.33)	(0.25)	0.48	M, C, R
*Multiple diagnoses indication of tracer drugs*								
Parkinson’s disease	914	2,219	0.67	0.99	(0.38)	(0.27)	0.66	
*Alternative refinement*								
w/o anti-psychotic	914	1,739	0.65	0.99	0.44	(0.37)	0.64	M, C, R
Schizophrenia and other disorders	1,911	6,439	0.67	0.95	(0.29)	(0.18)	0.64	
*Alternative refinement*								
w/o anti-cholinesterasic	1,911	5,985	0.66	0.96	(0.30)	(0.20)	0.64	C, R
Epilepsy	1,656	5,727	0.54	0.96	(0.23)	(0.14)	0.52	
*Alternative refinement*								
w/o chronic pain drugs	1,656	4,260	0.52	0.97	(0.28)	(0.19)	0.50	C, R
*Prophylactic treatment*								
Thrombo-embolic risk or disease	6,122	29,427	0.72	0.76	(0.18)	(0.10)	0.61	
*if including thrombogenic cardiac diseases*	17,804	29,427	0.73	0.82	0.45	(0.33)	0.63	M, C, R
Diseases of esophagus, peptic ulcer	3,805	31,331	0.72	0.73	(0.11)	(0.05)	0.60	^c^
**With all K values < 0.4**								
*Acute diseases with time limited treatment, minor or rare conditions as inpatients*								
Migraine	601	1,771	0.29	0.99	(0.14)	(0.09)	0.28	C
Vertigo	623	2,084	0.29	0.98	(0.13)	(0.08)	0.28	^c^
Bacterial infection or septicemia	10,433	41,704	0.58	0.64	(0.13)	(0.05)	0.32	^c^
Eye inflammation	467	6,827	0.33	0.94	(0.04)	(0.02)	0.29	C
Dermatitis	1,091	14,461	0.33	0.87	(0.03)	(0.02)	0.23	C , R
Mycosis	988	16,971	0.36	0.85	(0.03)	(0.01)	0.25	C
Other diseases of upper respiratory tract – allergy	466	9,917	0.31	0.91	(0.02)	(0.01)	0.24	C
Disorders of external ear	71	2,070	0.27	0.98	(0.02)	(0.01)	0.25	C
Parasitosis	61	1,984	0.26	0.98	(0.01)	(0.01)	0.25	C
Other acute upper respiratory infections	856	32,259	0.50	0.71	(0.01)	(0.01)	0.28	^c^
Acne	72	2,774	0.25	0.97	(0.01)	(0.01)	0.23	C , R
Psoriasis	466	1,188	0.35	0.99	(0.19)	(0.13)	0.34	C , R
Gout	659	2,126	0.39	0.98	(0.18)	(0.12)	0.38	C , R
Female inflammatory diseases	864	6,331	0.25	0.94	(0.05)	(0.03)	0.20	
Conjonctivitis	114	6,458	0.24	0.94	(0.01)	(0.00)	0.19	
Hemorrhoids	1,188	3,048	0.19	0.97	(0.10)	(0.06)	0.16	
Skin or subcutaneous infections	695	9,565	0.21	0.91	(0.02)	(0.01)	0.14	
Paludism 28 444 0.14 1.00 0.02 0.01 0.14	28	444	0.14	1.00	(0.02)	(0.01)	0.14	
Intestinal infectious diseases	1,608	7,131	0.19	0.94	(0.06)	(0.03)	0.13	
Viral diseases	1,261	1,801	0.15	0.98	(0.11)	(0.09)	0.13	
Influenza	177	74	0.00	0.00	(0.00)			
*Multiple diagnoses indication of tracer drugs*								
Inflammatory polyarthritis and connective tissue disorders	1,321	6,526	0.39	0.94	(0.12)	(0.07)	0.35	^c^
Anaemias – nutritional	3,122	11,845	0.31	0.90	(0.10)	(0.06)	0.23	^c^
Other mental and behavioral disorders	7,852	28,126	0.54	0.76	(0.16)	(0.08)	0.37	^c^
Pain	12,341	66,022	0.73	0.41	(0.07)	(0.02)	0.31	^c^
*Severe pain*^*d*^	12,341	7,429	0.16	0.92	(0.13)	(0.13)	0.07	
*Prophylaxis treatment*								
Tuberculosis	216	226	0.31	1.00	(0.30)	(0.29)	0.30	
*Active tuberculosis*	216	82	0.25	1.00	(0.36)	(0.66)	0.25	C, R
*Alternatives to pharmacological treatment*								
Neutropenia	468	369	0.18	1.00	(0.21)	0.26	(0.17)	
*Extended to prophylaxis of myelosuppressive chemotherapy*	1,452	369	0.16	1.00	(0.25)	0.63	(0.16)	C, R
Obesity	6,106	639	0.03	1.00	(0.05)	0.23	(0.02)	^c^
Other endocrine nutritional and metabolic diseases	8,393	160	0.00	1.00	(0.01)	0.19	(0.00)	
Hemorrhagic risk or disease	1,434	756	0.05	0.99	(0.07)	0.09	(0.05)	
Poisonings	1,061	309	0.00	1.00	(0.00)	0.00	(0.00)	
Heart failure	3,166	94	0.00	1.00	(0.00)	0.02	(0.00)	
*Alternative refinement: see hypertension/heart failure above*								
Abuse of tobacco	7,062	14	0.00	1.00	(0.00)	0.08	(0.00)	

^a^Kappa values, not used as judgment criteria are in parenthesis.

^b^Context of use: morbidity indicator (M), ambulatory costs control (C), insurers’ risk adjustment (R) only for chronic conditions.

^**c**^Appropriateness of pharmacologic treatment uncertain, prone to practice variations.

^d^Strong opioids.

Year-to-year category stability was measured by the Κ_1_ coefficient. We expected chronic diseases detected in 2005 to also be detected in 2006, as reflected by a high K_1,_ i.e. >0.6 [33].

Cohen Kappa coefficients were given with a 95% confidence interval [34].

## Results

Table 3 lists morbidity groups with corresponding ICD-10 and ATC codes. Eighteen diagnostic categories of the ICD shortlist were left unmodified, and 31 were subdivided to fit drug categories. Five morbidity groups were built by grouping diagnostic categories, and six by grouping subcategories. Thus we obtained 60 morbidity groups derivable from drugs dispensation. Morbidity groups were attributed independently to all insured inpatients from coded diagnoses (Table 3, ICD-10 column) and dispensed drugs (Table 3, ATC codes).

**Table 3:** Description of morbidity groups

**Morbidity groups**^**a**^	**ICD-10 codes**	**ATC codes**	**Codes added + or removed-**^**b**^
*Unmodified diagnostic categories*			
Tuberculosis (03)	A15A19,B90,K230,K673,K930,M011,M490,M900,N330,N740, N741,O980,P370	J04A	J01GA01- J01GB04-
Human immunodeficiency virus disease (05)	B20-B24,F024,R75,Z21	J05AE,J05AF,J05AG,J05AR, J05AX07-J05AX09 except J05AF08	J05AF05+
Diabetes mellitus (22)	E10-E14,G590,G632,H360, M141,N083,O24	A10	
Mental and behavioral disorders due to alcohol (25)	F10	N07BB	N07BB+
Schizophrenia, schizotypal and delusional disorders (27)	F20-F29	N05A , except N05AN	N05AD0+ N05AL0+
Mood [affective] disorders^c^ (28)	F30-F39	N06A,N06C,N05AN except N06AX01 and N06AX02	
Other mental and behavioral disorders^d^(29)	F04-F09,F40-F99 not F803	N05B,N05C,N06B	N05BD0+ N05BX0+ N05C + N06B+
Alzheimer’s disease^e^ (30)	F00,G30	N06D not N06DX02	
Multiple sclerosis* (31)	G35	L03AB02,L03AB07,L03AB08, L03AX13	
Epilepsy (32)	G40-G41,F803	N03	N03AA0+
Hypertensive disease (38)	I10-I15,O10-O11,O13-016	C02,C03 except C03C,C04AB, C07,C08,C09	C04AB + C07 + C08 + C09+
Conduction disorders and cardiac arrhythmias (43)	I44-I49	C01A,C01B,C01CA02,C01EB10	C01CA02+
Heart failure^f^ (44)	I50	C01CA04, C01CA07,C01CE,C01CX,C03C	C01CA04+ C01CX+
Crohn’s disease and ulcerative colitis (65)	K50-K51	A07EA*,*A07EC	A07EA+
Infections of the skin and subcutaneous tissue* (77)	L01,L02,L08	D06,P03	
Hyperplasia of prostate (94)	N40	G04BD,G04C	G04BD + G04C+
Inflammatory diseases of female pelvic organs* (97)	N70-N77, except N740 and N741	G01	
Poisoning by drugs and biological substances* (122)	T36-T65	V03AB,H04AA01	
*Subgroups of diagnostic categories*			
Paludism* (06-)	B50-B54	P01B not P01BA02	
Parasitosis* (06-)	B55-B83, B89	P01A,P01C,P02	
Hepatitis B or C (06-)	B16-B18	L03AB01,L03AB04,L03AB05,L03AB06,L03AB09,L03AB10, L03AB11, J05AF08, J05AF10, J05AF11	J05AB04- J05AF05- L03AB01+ L03AB06+ L03AB09+ L03AB11+
Viral diseases* (06-)	A60,A80-A99,B00-B15,B19, B25-B34,B941,B942,B97,J12, J171,J203-J207,J210	J05AA, J05AB, J05AC03, J05AD, J05AX except J05AX07-J05AX09	
Anemia – nutritional* (20-)	D50-D53	B03A, B03B	
Non nutritional anemia ^g^ (20-)	D55-64	B03XA	
Neutropenia* (21-)	D70	L03AA	
Thyroid disorders (23-)	E00-E07	H03	
Other endocrine, nutritional and metabolic diseases* (23-)	remainder of E00-E90	H01AB, H01AC, H01AX, H01C except H01CB01	
Obesity* (23-)	E66	A08	
Hyperlipidemia (23-)	E780-E785	C10	
Mental and behavioral disorders due to opioids* (26-)	F11,F19	N07BC	
Mental and behavioral disorders due to tobacco (26-)	F17	N07BA	N07BA+
Migraine (34-)	G43	N02C	N02C+
Parkinson’s disease (34-)	G20-G22	N04,except N04BC08	N04BC01+ N04BC06 + ^h^ N04BC07+
Conjunctivitis* (36-)	H10	S01A, S01GX	
Glaucoma (36-)	H40, H42	S01E	
Eye inflammation* (36-)	H00-H05,H11-H16,H20, H22	S01B, S01C	
Disorders of external ear* (37-)	H601-H609,H62	S02, S03	
Vertigo* (37-)	H81,H82	N07C	
Influenza* (49-)	J09-J11	J05AH, J05AC02	
Other acute upper respiratory infections* (49-)	J00-J06, not J020,J028,J030, J038	R01, R02, R05	
Other diseases of upper respiratory tract – allergy (53-)	J30,L50	R06, V01	R06 + V01+
Hemorrhoids* (69-)	I84, O224, O872	C05A	
Functional disorders of the digestive system* (76-)	K58, K59	A03, A06, A07DA, A07B, A07CA	
Psoriasis (78-)	L40	D05, L04AA21, L04AA15	D05 + L04AA21+ L04AA15+
Dermatitis* (78-)	L20-L39, not L303	D07, D11AX14, D11AX15	
Acne* (79-)	L70,L718,L719,L730	D10	
Gout (83-)	M10	M04A	
Osteoporosis (89-)	M80-M82	H05, M05BA, M05BB	H05 + M05BA + M05BB+
Transplanted organ status (129-)	K771,K932,L991,N165,T860, T861-T864,T8681,T8682,Z94	L04AA02-10, L04AX01, L04AC01, L04AC02, L04AD01, L04AD02	
*Multiple morbidities groups*			
Gastroenteritis of presumed infectious origin* (01–02)	A00-A09, not A064-A066, K521	A07A, A07F	
Ischemic heart diseases (39–41)	I20-I25	C01DA, C01DX12, C01DX16	C01DX12+
Reactive airway disease^i^ (54, 55)	J40-J47	R03	R03AA + R03AB + R03BZ + R03CA + R03CB+
Diseases of esophagus and peptic ulcer (59–61)	K20-K31, not K230 and K231	A02BA- A02BD, A02X02	
Malignant neoplasm (07–15)	C00-C97	A04AA, A04AD12, L01, L02, L03AC01, L03AX03, L03AX12 L04AX02, V03AF, V03AG, V03AH, V10A, V10B, V10XX01	L03AA-^m^ A04AD12+ L02+ L03AC01+,L03AX03+,L03AX12+ L04AX02 + V03AF + V03AG + V03AH + V10A + V10B + V10XX01+
Inflammatory polyarthritis and connective tissue disorders (83,85-)^j^	M05-M09, M30-M36, M45	H02A, H02B,L04AA13,L04AB01, L04AB04, L04AX03, M01B, M01CA, M01CB, P01BA02	L04AX03+ M01B + M01CA+
Pain (80-83-,85-88-,111,112-)^k^	M50-M51,M54,R07,R10, R30, R51, R52, remainder of M60-M79	M01A, M03B, M03C, M09, N02A, N02B	M03B + M03C + M09+ N02B+
Mycosis (06-,50-)*	B35-B49, J172	D01, J02	
Bacterial infection or septicemia* (04,06-,50-,51-,88-)	A064-A066, A20-A79 (except A30, A31, A60), B95,B96, B99, D733, E321, G00, G01, G042, G060-G062, G07, H600, J020, J028, J030, J038, remainder of J13-J20, J36, J390, J391, J851-J853, K046, K047, K1020, K1021, K113, K122, K351, K570, K572, K574, K578, K61,K630, K750, L00, L03-L05, M00, M010-M013, M03, M600, M630-M632, M650, M651; M680, M710, M711, M730, M731, N151, N160, N340, N412, N450, N459, N751, N764, O85, O911	J01	
Thrombo-embolic risk or disease (34-, 42-,45-,46-,48,101-,102-,106-)^l^	G08, I260,I269,I2720, I63-I66,I676, I693, I694, I698, I70, I731,I74, I77, I80-I82, O032, O037, O042, O047, O052, O057, O062, O067, O072, O077, O082, O223, O225, O871, O873	B01A	
Hemorrhage risk or disease* (21-, 45-,46-)	D65-D68, D693-D699	B02,L03AC02	

^a^The numbers identifying groups are those of the International Shortlist for Hospital Morbidity Tabulation (ISHMT) - Eurostat/OECD/WHO; with dash indicating subgroups. Categories not included in the most recent RxRisk model^18^ are indicated with an asterisk*.

^b^Codes removed or added to those of Kuo et al.^14^

^c^The RxRisk-V distinguishes bipolar disorders (ATC code N05AN) and depression.

^d^This category is defined as anxiety and tension in the RxRisk-V (hypnotics not retained as screen).

^e^This category is defined as dementia in the RxRisk-V.

^f^The RxRisk-V split hypertension in hypertension and congestive heart failure/hypertension, the latter detected by loop diuretics, Angiotensin converting enzyme inhibitors and Angiotensin II receptors blockers.

^g^Defined as end stage renal disease in the RxRisk-V.

^h^ATC distinguishes, according the dose, the use of the two dopamine agonists bromocryptin and cabergolin N04BC01 , N04BC06: low dose for prolactin inhibition and high dose in parkinsonism.

^i^Asthma, chronic obstructive pulmonary disease and bronchiectasis.

^j^Rheumatic conditions for original CDS and steroid responsive conditions for Rxrisk.

^k^The RxRisk-V distinguishes pain(opioid) from pain and inflammation (NSAI).

^l^The RxRisk-V distinguishes anti-platelet from anticoagulant.

^m^In our category neutropenia.

The studied population included 108,915 insured enrollees followed throughout 2005 and 2006; they were hospitalized at least once, and did not obtain over 5% of their drugs via self- dispensation by a physician. Sixty four percent of hospitalizations (N = 70,083) were classified in at least one of the morbidity groups listed in Table 3 (average number of categories: 2.75). Eighty percent (N = 86,915) took a drug associated with at least one of these morbidity categories (average number of categories: 6.08). The mean age of the studied population was 53.1 years (SD 22.5), with a 44% proportion of men. Results for the 60 morbidity groups identified by drugs or hospitals diagnoses are given in Table 2. All K_c_ confidence intervals were narrow, i.e. not more than 1% above or below the estimates.

Five morbidity categories (transplant, diabetes, HIV disease, hypertension and thyroid disorders) had a K_c_ exceeding 0.4, justifying the use of drug-based information for morbidity indicators, cost control and risk adjustment purposes. As most antihypertensive agents are also recommended for heart failure, we combined those two categories and obtained an enhanced agreement between drugs and diagnoses.

Eight conditions can be treated without drugs (false negatives), justifying a K_0_ greater than 0.4: no indication of long-term treatment for progressive multiple sclerosis, non-nutritional anemia, or chronic hepatitis, surgery for ischemic heart disease, conduction disorders, or malignant disease, and the psychosocial treatment of alcohol or opioid abuse. Considering chronic renal failure and chemotherapy as proxies of non-nutritional anemia (usual complications), we observed a significantly increased K_0_ (0.63), suggesting a possible under-coding of the condition. All these conditions identified from drug dispensations correspond to actual morbidities, justifying their use for cost control and risk adjustment.

Five conditions were related to obvious under-coded diagnoses with a K_1_ over 0.4: glaucoma, hyperlipidemia, functional digestive disorders, prostate hyperplasia, and osteoporosis. These conditions are often not coded because they seldom merit hospital treatment. We also found evidence of under-coding for four other morbidities with K_1_ >0.4, as shown by the improvement of K_0_ to fair values when the screen is restricted to those subjects who receive the most treatment, and were thus more likely to feature in the hospital data. Indeed, K_0_ increased from 0.18 to 0.36 for mood disorders when the screen was restricted to patients taking three classes of psychotropic drugs; from 0.25 to 0.46 for reactive airway disease (RAD) when screening criteria required inhaled and systemic corticoids; from 0.31 to 0.43 for Alzheimer’s disease when criteria included memantine use (indicated for more severe impairments); from 0.25 to 0.33 for inflammatory bowel diseases (IBD) when criteria included systemic corticoid or immune-suppressors in addition to the tracer drugs.

Three conditions were difficult to detect because some tracer drugs can be prescribed for other diseases, explaining false positives and the use of K_1_ value as criteria. The following refinements of the algorithms significantly improved K_c_ values without excessively lowering K_1_ values (see Table 2): removing the association of an anti-cholinergic medication and a neuroleptic from the screen of Parkinson’s disease (treatment of neuroleptic-induced extra-pyramidal symptoms); removing the association of neuroleptic and anti-cholinesterasic drugs from the screen of psychotic disorders (treatment of behavior disorders of dementia); removing the association of opioids with gabapentine or pregabaline from the screen of epilepsy (treatment of chronic pain). There was also some evidence of under-coding of epilepsy, because K_o_ increased to 0.35 when we restricted the screen to patients with multiple antiepileptic drugs.

Two morbidity groups, for which tracer treatment is mainly preventive (thromboembolism risk or disease, and diseases of esophagus/peptic ulcer), had high K_1_ but very low K_0_ (<=0.10). Extending ischemic heart disease and thrombogenic cardiac arrhythmias to thrombo-embolic disease increased all Kappa values, suggesting that secondary prevention is often the treatment aim. Enhancement in accuracy of peptic disease screening by removing patients taking a non steroidal anti-inflammatory (primary prevention hypothesis) was negligible.

Thirty two morbidity categories exhibited poor fit between diagnoses and drugs. Most of them were acute conditions requiring time-limited drug treatment or minor conditions seldom occurring or collected in hospitals. We considered that if such conditions (migraine, mycosis, acne for instance) had K_1_ >0.2, they corresponded to an actual morbidity, but had not been recorded in the hospital data. However, the uncertainty surrounding the appropriateness of treatment precluded the adoption of several groups in spite of fair K_1_ (see Table 2, last column). The same limitation prompted cautious use of most categories detected by tracer drugs that have multiple indications reaching acceptable K_1:_ hypnotics, painkillers, nutritional supplements.

At the end of the analysis, 15 morbidity groups were retained for morbidity indicators (Table 2, letter M), to which 16 were added for insurers’ risk adjustment (letter R) and six further groups for ambulatory cost adjustment (letter C). Twenty three were not retained because of their poor accuracy or their treatment was prone to practice variations.

Table 4 shows the reproducibility of drug information based morbidity categories from one year to the next. Chronic conditions, for which drug-based information performed the best were also more reproducible. All acute conditions had poor reproducibility.

**Table 4:** Stability of categories inferred from drugs on patients classified in at least one category in 2005 (N = 410,469)

**Morbidity categories**	**Detected in 2005**	**Detected in 2006**	**Detected both years**	**K**_**1**_
Diabetes mellitus	22,530	29,296	0.90	0.90
Transplant	914	1,183	0.88	0.88
Human immunodeficiency virus [HIV] disease	1,057	1,471	0.88	0.88
Multiple sclerosis	353	496	0.84	0.84
Hypertensive disease/heart failure	110,648	136,262	0.87	0.80
Hyperlipidemia	53,593	68,177	0.83	0.80
Glaucoma	9,413	12,019	0.79	0.78
Thrombo-embolic risk or disease	59,070	73,837	0.78	0.73
Osteoporosis	11,272	14,056	0.72	0.71
Thyroid disorders	9,485	11,271	0.70	0.69
Alzheimer’s disease	2,158	2,730	0.69	0.69
Schizophrenia, schizotypal and delusional disorders	12,216	14,522	0.65	0.64
Parkinson’s disease	4,021	4,885	0.64	0.64
Epilepsy	9,290	11,432	0.64	0.63
Malignant neoplasm	5,324	7,553	0.63	0.62
Gout	5,283	6,715	0.62	0.61
Crohn's disease and ulcerative colitis	1,999	2,417	0.59	0.58
Anemia - others	555	801	0.57	0.57
Mood [affective] disorders	52,395	60,280	0.63	0.56
Conduction disorders and cardiac arrhythmias	3,959	4,734	0.56	0.56
Hyperplasia of prostate	12,835	15,751	0.56	0.55
Ischemic heart disease	7,641	7,417	0.49	0.48
Migraine	7,297	7,891	0.49	0.48
Other mental and behavioral disorders	73,679	82,593	0.58	0.47
Other endocrine, nutritional and metabolic diseases	653	778	0.47	0.47
Abuse of opioid	58	74	0.43	0.43
Reactive airway disease	39,237	44,497	0.48	0.42
Diseases of esophagus and peptic ulcer	69,660	83,280	0.52	0.40
Hepatitis B or C	223	260	0.31	0.31
Obesity	1,497	2,196	0.31	0.30
Psoriasis	4,042	4,357	0.31	0.30
Mental and behavioral disorders due to alcohol	979	916	0.29	0.29
Vertigo	5,852	5,523	0.25	0.24
Acne	14,031	15,284	0.27	0.24
Anemia - nutritional	20,758	21,524	0.27	0.22
Functional digestive disorders	69,988	74,141	0.36	0.22
Neutropenia	174	280	0.22	0.22
Inflammatory polyarthritis and connective tissue disorders	12,934	12,965	0.24	0.22
Poisonings	438	763	0.21	0.20
Viral diseases	4,606	5,338	0.20	0.18
Mycosis	51,825	51,635	0.27	0.17
Other diseases of upper respiratory tract - allergy	32,151	32,610	0.23	0.17
Inflammatory diseases of female pelvic organs	17,599	16,556	0.20	0.16
Hemorrhoids	7,991	6,973	0.17	0.16
Tuberculosis	111	72	0.14	0.14
Eye inflammation	17,078	17,985	0.18	0.14
Dermatitis	49,684	47,769	0.23	0.13
Conjunctivitis	24,435	25,155	0.18	0.13
Abuse of tobacco	53	60	0.09	0.09
Infections of the skin and subcutaneous tissue	29,384	29,508	0.15	0.09
Gastroenteritis of presumed infectious origin	21,755	18,053	0.13	0.09
Hemorrhagic risk or disease	1,476	1,267	0.08	0.08
Disorders of external ear	8,967	7,396	0.08	0.07
Other acute upper respiratory infections	134,478	130,051	0.36	0.06
Paludism	2,270	2,233	0.06	0.06
Parasitosis	5,435	4,718	0.07	0.05
Influenza	314	105	0.02	0.02
Bacterial infection or septicemia	132,933	139,298	0.32	0.00
Pain	235,400	257,672	0.53	0.00

Morbidity prevalence (crude and adjusted) inferred from 2005 drug information for the whole insured population (N = 2,028,620, mean age 55.5, SD age 22.7, men 39.3 percent) are shown in Table 5 and compared with available national values or estimates from other external sources. All of our estimates were fairly close to the reference estimates, with only a few exceptions, Parkinson and transplants were overestimated (by a factor of 2 and 3 respectively), whereas HIV, Alzheimer’s disease, prostate hyperplasia and osteoporosis were underestimated by a factor between 2 and 4.

**Table 5:** 2005 estimated prevalence of illness inferred from drugs’ dispensation among 2,028,621 insured

**Morbidity categories**	**Crude rates (%)**	**Gender and age adjusted rates (%)**	**Published prevalence rates (%)**^**b**^
Pain	31.6	31.4	
Other acute upper respiratory infections	18.1	18.2	
Bacterial infection or septicemia	17.5	17.4	
Hypertensive disease/heart failure	14.9	19.0	Aged 35–75: 36.7, 50.1% currently treated [35]
Mental disorders	14.1	14.1	Aged 18–65: 26.0 -28.3 [36]
Other mental and behavioral disorders	10.1	9.3	
Mood [affective] disorders	7.5	8.3	Major depression: 2-7 [36,37]
Schizophrenia, schizotypal and delusional disorders	1.7	1.8	0.2–1.3 [36,37]
Diseases of esophagus and peptic ulcer	9.4	9.1	
Functional digestive disorders	9.2	9.0	
Thrombo-embolic risk or disease	7.9	7.4	
Mycosis	7.2	7.1	
Hyperlipidemia	7.3	10.8	Aged 35–75 treated: 13.5 [35]
Dermatitis	6.4	6.3	
Other diseases of upper respiratory tract - allergy	5.3	5.4	
Reactive airway disease	5.4	5.6	Aged > 29: 5.0 [38]
Infections of the skin and subcutaneous tissue	3.9	3.9	
Conjunctivitis	3.2	3.3	
Diabetes mellitus	3.1	3.8	Aged >19: 3.5-4.3 [39,40]
Gastroenteritis of presumed infectious origin	2.8	2.8	
Anemia - nutritional	2.6	2.5	
Inflammatory diseases of female pelvic organs	2.5	2.5	
Eye inflammation	2.1	2.1	
Hyperplasia of prostate	2.2	5.2	Males > 39: 13-28 [41]^,a^
Acne	1.8	1.9	
Inflammatory polyarthritis and connective tissue disorders	1.7	1.6	
Glaucoma	1.6	2.9	Aged > 39: 2 [42]^,a^
Osteoporosis	1.5	3.3	Aged > 39: 13.8 [43]
Epilepsy	1.3	1.3	1.0 [44]^,a^
Thyroid disorders	1.2	1.2	1.0 [45]^,a^
Disorders of external ear	1.1	1.1	
Hemorrhoids	1.0	1.0	
Migraine	0.99	0.98	
Ischemic heart disease	0.99	0.89	
Malignant neoplasm	0.74	0.71	
Vertigo	0.73	0.70	
Parasitosis	0.68	0.68	
Gout	0.70	0.66	
Viral diseases	0.61	0.60	
Psoriasis	0.53	0.52	
Parkinson’s disease	0.54	1.68	Aged > 59: 0.91 [46]
Conduction disorders and cardiac arrhythmias	0.52	0.47	
Paludism	0.29	0.29	
Alzheimer's disease	0.30	1.26	Aged > 59: 4.5 [46]
Crohn's disease and ulcerative colitis	0.27	0.26	0.21 [47]
Hemorrhagic risk or disease	0.19	0.24	
Obesity	0.22	0.21	
Human immunodeficiency virus [HIV] disease	0.15	0.14	0.3 [48]
Transplant	0.12	0.12	Renal grafts only ~0.04 [49]^,b^
Mental and behavioral disorders due to alcohol	0.12	0.12	
Other endocrine, nutritional and metabolic diseases	0.09	0.09	
Anemia - others	0.08	0.08	
Poisonings by drugs, medication and biological substances and toxic effects of substances chiefly nonmedicinal	0.06	0.06	
Multiple sclerosis	0.05	0.05	
Influenza	0.04	0.04	
Hepatitis B or C	0.03	0.03	
Neutropenia	0.03	0.02	
Tuberculosis (active)	0.01	0.01	0.008 [50]
Abuse of opioids	0.01	0.01	

^a^International data due to lack of Swiss data.

^b^Considering 250 transplants per year between 1998 and 2006, and a graft half-life of 13 years.

Only 15% of all prescriptions (i.e. a substance identified by its ATC code delivered to a patient, N = 3,185,997) were not assigned to a morbidity category; these were mainly vitamins and other food supplements (20%), topic medications (36%), vaccines and immune globulin preparations (10%), contraceptives and hormonal replacement therapy (11%), anesthetics (2%), blood products and replacement fluids (2%).

## Discussion

Our drugs-based morbidity groups include the majority of chronic categories of the most recent CDS derived tools [12-19]. The few CDS categories our classification ignored were those deduced from devices or non-pharmaceutical prescriptions (urinary incontinence, ostomy, neurologic bladder, malnutrition), and three with poor screening (pancreatic insufficiency, hyperkaliemia and liver failure, the latter removed by other authors due to different indications of amonemia detoxicants in many countries) [17]. A few chronic illnesses and seventeen acute conditions were added. Although we retained most ATC codes of the revised CDS of Kuo and al, many drugs were added and some removed [14].

Fifteen chronic conditions (Table 2, M in last column) exhibited sufficient agreement with their diagnoses-based counterparts to be considered alternative strategies to diagnosis for capturing similar populations. Furthermore, all chronic conditions but four (IBD, mood disorders, prostate hyperplasia and RAD) exhibited substantial or almost perfect reliability on test-retest procedure. Finally, prevalence estimates largely agreed with estimates from national epidemiological studies whenever prevalence information is available in Switzerland. Similar age and gender distribution was found for diabetes [39,40], treated hyperlipidemia [35,51], treated hypertension [35,52], IBD [47] and reactive airway disease [38]. Our HIV disease prevalence agrees with the national estimate of 0.3%, in view of the fact that 75% of subjects registered in the Swiss HIV cohort and thus subject to close follow-up and compulsory treatment receive an antiretroviral therapy [53]. The moderate reproducibility of IBD, mood disorders and RAD may reflect the fact that therapy varies depending on the severity of conditions like these, which are characterized by exacerbation and remission periods [54]. Regarding prostate hypertrophy this may reflect failed treatment and a switch to surgery [55].

For nine conditions (see Table 2, K_o_ > 0.4 and indicated in the last column by C, R), drug-based information accurately identified only a subset of the population defined by diagnoses. These drugs are often used in a specialist care setting (e.g. chronic hepatitis, malignant neoplasm, multiple sclerosis, neutropenia) and thus might detect individuals with special or more costly care needs. Most conditions had only fair or moderate test-retest reliability reflecting time-limited treatment. Although their ability to describe the distribution of illnesses is poor, these categories fit medical conditions sufficiently well to be used to analyze medical practices and risk adjustment, providing better identification of the severity of a disease than only inpatient diagnoses.

Restricting tuberculosis screening to active cases - i.e. treated by more than one therapeutic agent - enhanced accuracy; low sensitivity may be due to time-limited therapy. However, our estimated prevalence agrees with the national estimates [50].

For all other conditions, agreement between hospital diagnoses and drug information was poor, which does not mean that information inferred from drugs is useless. The relationship between drug and diagnoses-based screening is not straightforward and would benefit by closer examination. Epilepsy and psychotic disorders are two examples where diagnoses- based conditions are much less frequent than drug-based conditions, but the interpretation is likely to differ. For epilepsy, where treatment is often continued several years after the last seizure, many subjects might have treatment renewed without a coded diagnosis. In such a case, drug-based information could offer a more complete overview of the condition. On the other hand, it seems unlikely that the diagnosis will be overlooked for psychotic disorders. Because they are used to treat other severe psychiatric disorders, antipsychotic drugs cannot be consistently associated with overlooked psychotic disorders.

Categories in which drug information detects a greater number of conditions than diagnosis pose a particular problem, since therapy might more effectively reflect medical practices than true conditions. For instance, primary prevention of peptic ulcer by means of nonsteroidal anti-inflammatory drugs does not provide evidence of an active disease, and therapy for functional dyspepsia may be disputable. If the acute nature of bacterial infection may explain the poor fit between diagnoses and drug-based information, the fact that antibiotherapy is often prescribed inappropriately is an important aspect when considering pharmacy-based screening. The high prevalence of other broadly defined conditions, e.g., pain, certain mental and behavioral disorders may also highlight their overestimation.

While some drugs-based morbidity groups were very specific (i.e. tuberculosis, vertigo, psoriasis, neutropenia), others were broad (viral diseases, malignant neoplasms). For the latter, more accurate information could be obtained from other data sources, including cancer registries. Missing information due to hospital drug dispensation might also explain the poor sensitivity of malignant neoplasm.

The most widespread application of disease status measure computable from routinely available data is to correct for confounding when comparing health care service indicators. Which rate of error is tolerable when estimating a population’s health from drugs dispensation depends on the purpose of the indicator [1]. Morbidity indicators require satisfactory agreement between drugs-based and diagnoses categories (see Table 2). The basis of this interpretation is the Kappa thresholds defined by the authors**.** Only 15 categories fit this purpose. For other indicators, such a stringent criterion is not required. Inpatient diagnoses have limited pertinence, as only a minority of enrollees is hospitalized, particularly under age 65. Consequently, some data are better than none, but only if the measure creates no perverse incentives. Several categories that reflect medical practices rather than true morbidity must be viewed with caution, even when they have good predictive performance (overuse of medication).

The main limitation of our study is that we restricted the validation of drug-based classification to inpatients, thus underestimating specificity by under-reporting chronic co-morbidities. On the other hand, sensitivity is measured on a population suffering from more severe conditions and might be overestimated. There might be variations in the hospital coding practices across countries due to differences in coding rules, coding purposes such as hospital payment or the thoroughness of secondary diagnosis coding, which is sometimes limited by the data fields available. Therefore, caution should be exercised when generalizing our results to other countries. Some conditions, mainly those treated in ambulatory settings, are poorly represented. Further studies conducted in ambulatory settings might rate drugs-based acute or milder categories differently. Another problem is that we may be identifying incidental users of a drug rather than a real condition; for example, having been treated by a proton-pump inhibitor or a painkiller does not mean having a disease.

## Conclusion

We defined sixty morbidity categories that may in theory be related to a particular drug signature that might be applied internationally. Drug information was a good proxy of diagnoses to identify 15 chronic conditions, providing useful information for epidemiological studies. Although the accuracy of detection was only fair, twenty-two other morbidities could also be exploited for health insurers’ risk adjustment or ambulatory cost control. Several categories were excluded because they prone to variations in prescribing (pain, bacterial infection, peptic ulcer). Some acute diseases poorly represented in hospitalized populations should be studied further on outpatient samples. Further research should also focus on more detailed validation, e.g. using medical records or other more precise data.

## Abbreviations

EMR: Electronic medical records; WHO: World Health Organization; ATC: Anatomical Therapeutic Chemical; CDS: Chronic disease score; SFSO: Swiss Federal Statistical Office; ICD-9-CM: International Classification of Diseases, Ninth Revision, Clinical Modification; ICD-10: International Statistical Classification of Diseases, Tenth Revision; ISHMT: International Shortlist for Hospital Morbidity Tabulation; K: Kappa coefficient; HIV: Human immunodeficiency virus; RAD: Reactive airway disease; IBD: Inflammatory bowel disease.

## Competing interests

The authors declare that they have no competing interests.

## Authors’ contributions

ES prepared data and participated in the statistical analysis. AD reviewed the correspondence between drugs and diagnoses based groups. PH and YE designed and conducted the study, and drafted the manuscript. All authors have read and approved the final manuscript.

## Pre-publication history

The pre-publication history for this paper can be accessed here:

http://www.biomedcentral.com/1472-6963/13/453/prepub

## Supplementary Material

Additional file 1 **Appendix A.** Morbidity groups that cannot be inferred from drug dispensations. Appendix B. Drugs that did not screen for specific morbidities (ATC codes). Appendix C. Weighted Kappa. Click here for file
