# Supplementary material for: Disease identification based on ambulatory drugs dispensation and in-hospital ICD-10 diagnoses: a comparison
Source: BMC Health Serv Res. 2013 Oct 31;13:453. doi: 10.1186/1472-6963-13-453 (PMC4228448; doi:10.1186/1472-6963-13-453)
Supplement: Additional file 1 — Appendix A. Morbidity groups that cannot be inferred from drug dispensations. Appendix B. Drugs that did not screen for specific morbidities (ATC codes). Appendix C. Weighted Kappa. [file 1472-6963-13-453-S1.doc]

**Appendix A. Morbidity groups that cannot be inferred from drugs dispensation**

ISHMT Morbidity groups ICD-10 codes
number*

100-107 Obstetrics conditions O00-O99

108,110,128 New born P00-Q99, Z38

115-121 Trauma S06-T14,T20-T32,T79

127 Contraceptive management Z30

*Surgical groups:*

35 Cataract H25-H26, H28

52 Chronic diseases of tonsils and adenoids J35

62 Diseases of appendix K35-K38

63 Inguinal hernia K40

64 Other abdominal hernia K41-K46

67 Paralytic ileus and intestinal obstruction without hernia K56

68 Diverticular disease of intestine K57

73 Cholelithiasis K80

80 Coxarthrosis [arthrosis of hip] M16

81 Gonarthrosis [arthrosis of knee] M17

82 Internal derangement of knee M23

92 Urolithiasis N20-N23

96 Disorders of breast N60-N64

99 Other disorders of the genitourinary system remainder of N00-N99

*Unspecified morbidities or symptoms:*

98 Menstrual, menopausal, and other female genital conditions N91-N95

113 Unknown and unspecified causes of morbidity (incl. those without a diagnosis) R69

114 Other symptoms, signs and abnormal clinical and laboratory findings remainder of R00-R99

123 Complications of surgical and medical care, not elsewhere classified T80-T88

124 After-effects of injuries, of poisoning and of other consequences of external causes T90-T98

125 Other and unspecified effects of external causes remainder of S00-T98

126 Medical observation and evaluation for suspected diseases and conditions Z03

129 Other medical care (including radiotherapy and chemotherapy sessions) Z51

130 Other factors influencing health status and contact with health services remainder of Z00-Z99

*Morbidities without specific drug treatment*

24 Dementia remainder of F01-F03

33 Transient cerebral ischaemic attacks and related syndromes G45

47 Varicose veins of lower extremities I83

56 Other diseases of the respiratory system J60-J99

57 Disorders of teeth and supporting structures K00-K08

58 Other diseases of oral cavity, salivary glands and jaws K09-K14

66 Other noninfective gastroenteritis and colitis K52

71 Alcoholic liver disease K70

74 Other diseases of gall bladder and biliary tract K81-K83

75 Diseases of pancreas K85-K87

84 Systemic connective tissue disorders M30-M36

90 Glomerular and renal tubulo-interstitial diseases N00-N16

91 Renal failure N17-N19

93 Other diseases of the urinary system N25-N39

95 Other diseases of male genital organs N41-N51

16-19 Benign neoplasms D00-D48, D90

99 Other urinary diseases remainder of N43-N49

*Remainder codes, without specific drug treatment*

06-oth Other infectious and parasitic diseases A30, A31, B85-B88, B91, B92, B940, B948, B949, K231

21-oth Other diseases of the blood and bloodforming organs
 and certain disorders involving the immune mechanism D69, D71-D89

26-oth Other abuses F12-F16, F18

34-oth Other diseases of the nervous system remainder of G00-G99

36-oth Other diseases of the eye and adnexa remainder of H00-H59

37-oth Diseases of the ear and mastoid process remainder of H60-H95

45-oth Cerebrovascular diseases remainder of I63-I69

48-oth Other diseases of the circulatory system remainder of I00-I99

50-oth Pneumonia remainder of J12-J18

51-oth Other acute lower respiratory infections remainder of J20-J22

53-oth Other diseases of upper respiratory tract J30-J34, J36-J39

69-oth Diseases of anus and rectum K60-K62

72-oth Other diseases of liver K71-K77

76-oth Other digestive diseases remainder of K55 to K93

78-oth Papulosquamous disorders L41-L45

79-oth Other diseases of the skin and subcutaneous tissue remainder of L00-L99

83-oth Other arthropathies M031-M036, M11-M124, M128-M141,
 M18-M21, M24-M25

85-oth Deforming dorsopathies and spondylopathies M40-M44, M46-M49

89-oth Other disorders of the musculoskeletal system and connective tissue remainder of M00-M99

*International Shortlist for Hospital Morbidity Tabulation (ISHMT) - Eurostat/OECD/WHO.

Abbreviation: oth, other.

**Appendix B. Drugs that did not screen for specific morbidities (ATC codes)**

Food supplements Vitamins (A11), mineral supplements (A12), enzymes (M09AB), food products (V06)

Blood products Blood products (J06), solution for perfusion (B05)

Hospital treatments anesthesia (N01), pulmonary surfactants (R07AA), therapeutic radioactive products (V10),

Diagnostic support Ophtalmic preparations (S01), diagnostic products (V04), contrast products (V08) and other radiodiagnostic products (V09)

Dermatological preparations Emollients (D02), wound (D03, D09), antipruriginous (D04), antiseptics and disinfection (D08), other
 (D11)

Preventive drugs Vaccins (J07), contraceptives and sexual hormones (G03)

Non specific treatments non specific treatment of digestive tract (A1, A02A, A04, A05A and B, A07X, A16), circulatory system (C04, C05B, C05C), uro and gynecology (G02, G04), other systems (M03, N07A, R07B, V03,V07)

**Appendix C. Weighted Kappa**

Assuming the notation defined in Table 1, the expected agreement e can be expressed as follows

e = (a+c) (a+b) + (c+d) (b+d) = a^2^+ab+ac+bc+bc+cd+d^2^+bd = a(a+b+c) + 2bc + d(b+c+d) = a(1-d) + 2bc + d(1-a) = a+d-2ad+2bc

Thus, K_c_=(o-e)/(1-e) = (a+d-a-d+2ad-2bc) / (P’Q + PQ’) =2(ad-bc)/(P’Q + PQ’)

We can note that K_c_ is a particular case of K_w_=(ad-bc)/(wPQ’ + (1-w)P’Q), with w expressing alternative weights

w=0.5 when K_w_=K_c_ i.e equally emphasis is placed on avoiding false negatives and false positives

w=0 if false negatives have zero utility
w=1 if false positives have zero utility
